# Prevalence, associated factors, and temporal variation of allergic rhinitis among 13 to 14-year-old adolescents from rural Sri Lanka: An analytical cross-sectional study

**DOI:** 10.5415/apallergy.0000000000000252

**Published:** 2026-01-13

**Authors:** Thiweda Subhanee, Sajeewa Thennakoon, Periyasami Sivabalan Sridharan, Tharusha Chamanthi Siriwardhana, Savithri Sulakkhana, Vimansha Sumanapala, Lahiru Wijayarathna, Janith Warnasekara, Shashanka Rajapakse

**Affiliations:** 1 Faculty of Medicine and Allied Sciences, Rajarata University of Sri Lanka, Anuradhapura, Sri Lanka; 2 Department of Physiology, Faculty of Medicine and Allied Sciences, Rajarata University of Sri Lanka, Anuradhapura, Sri Lanka; 3 Department of Community Medicine, Faculty of Medicine and Allied Sciences, Rajarata University of Sri Lanka, Anuradhapura, Sri Lanka; 4 Department of Public Health and AI, Graduate College of Cancer Science and Policy, National Cancer Centre, Goyang-si, Gyeonggi-do, South Korea; 5 Department of Physiology, Faculty of Medicine and Allied Sciences, Rajarata University of Sri Lanka, Anuradhapura, Sri Lanka

**Keywords:** Allergic rhinitis, adolescent health, epidemiology, public health

## Abstract

**Background:**

Allergic rhinitis is often underdiagnosed despite its high prevalence and considerable impact on academic achievement and quality of daily living. The study aims to describe the epidemiological patterns of allergic rhinitis among adolescents aged 13–14 years in Anuradhapura, Sri Lanka.

**Methods:**

An analytical cross-sectional study was conducted in 2023 among adolescents from 32 grade 8 classes in 6 government secondary schools located in the Anuradhapura municipal council area and randomly selected via multistage sampling. The prevalence and associated factors were assessed using the validated and translated International Study of Asthma and Allergy in Childhood questionnaire.

**Results:**

The study sample included 1,029 participants, of whom 528 (51.3%) were males. The prevalence of lifetime and current allergic rhinitis and eye symptoms was 37.6% (n = 387; 95% confidence interval [CI], 34.6–40.6), 25.9% (n = 266; 95% CI, 23.2-28.6), and 13.8% (n = 142; 95% CI, 11.8-16.0), respectively. Nasal symptoms negatively affected the activities of daily living in 195(18.9%; 95% CI, 16.6-21.5) adolescents, with moderate-to-severe disruption reported by 54 of them (20.3%; 95% CI, 15.6-25.6). Nasal symptoms were more common from October to February, with monthly precipitation significantly affecting the temporal variation (*P* = 0.003). Severe asthma (*P* < 0.01, adjusted odds ration [OR] 5.75; 95% CI, 3.79-8.72) was independently associated with allergic rhinitis, while sleeping on a rubber-mixed-coir mattress (adjusted OR 1.61; 95% CI, 0.95-2.74), and playing 4–6 times per week (adjusted OR 1.50; 95% CI 0.98-2.29) had higher odds for allergic rhinitis.

**Conclusions:**

Over a quarter of adolescents had current allergic rhinitis, and their symptom exacerbation followed a temporal pattern predicted by monthly precipitation. Severe asthma was an independent determinant of allergic rhinitis.

## 1. Introduction

Allergic rhinitis is a type I hypersensitivity reaction that is mediated by immunoglobulin E in response to allergen exposure, leading to inflammation of the nasal mucosa [1, 2]. This condition is characterized by a range of nonspecific symptoms, including sneezing, itching, nasal congestion, and rhinorrhoea, and is commonly accompanied by concurrent eye symptoms [2, 3]. It is the most prevalent noninfectious rhinitis across the globe, affecting approximately 400 million individuals [4]. The prevalence of allergic rhinitis is influenced by age and geography [5]. The prevalence of the disease is underestimated by the lack of recognition of symptoms and its non-life-threatening nature. However, it has been noted that identification of allergic rhinitis cases has been significantly increased over the past 20 years, becoming one of the top conditions for primary care visits [1, 6]. Moreover, this condition carries a high economic burden [7]. Failure to treat, undertreatment, or nonadherence to treatment increases direct and indirect costs, including costs associated with comorbidities such as sinusitis and asthma [7, 8].

Allergic sensitization and seasonal rhinitis are uncommon in children younger than 2 years, because it often takes at least 2 years of exposure to environmental allergens for the development of hypersensitivity [9]. The prevalence of seasonal allergic rhinitis in children aged 3 to 12 years is gradually rising annually at an approximate rate of 2% [10, 11]. The global prevalence of current rhinoconjunctivitis symptoms among adolescents aged 13 to 14 years is 14.6% [12]. The prevalence of the atopic triad, allergic rhinitis, asthma, and atopic dermatitis, is increasing, particularly in low to middle-income countries, affecting 30% of the population [13]. Current evidence suggests that allergic rhinitis is also highly prevalent in children and adolescents, affecting 25% of the adolescent population [14]. The prevalence rates of allergic rhinitis and rhinoconjunctivitis in the Indian subcontinent were high among adolescents aged 13 to 14 years, with 24.4% and 10.9%, respectively [15]. Limited studies conducted in Sri Lanka indicate that the prevalence of allergic rhinitis is common in both urban and rural populations [16]. However, epidemiological evidence of allergic rhinitis in Sri Lanka, especially among adolescents, is lacking.

Allergic rhinitis significantly impacts adolescents’ quality of life, causing sleep disturbance, impaired academic performance, and adversely affecting social and familial relationships [7]. The negative impact of allergic rhinitis during adolescence is critical because it is a crucial age for physical, cognitive, emotional, and social development, including puberty, brain development, identity formation, and relationships with peers and family members [17]. Globally, in adolescents aged 13 to 14 years, allergic rhinitis causes an estimated 2 million missed school days annually, significantly impairs academic performance, and causes persistent daytime fatigue and poor cognitive function [4, 18]. The epidemiology of allergic rhinitis, including contributing factors among adolescents from rural districts, is not adequately studied, hindering the implementation of targeted healthcare policies and practices to mitigate exposure to local risk factors. Therefore, this study aims to report the epidemiology and associated factors of allergic rhinitis and the monthly variation of symptoms among adolescents aged 13 to 14 years from the municipal council area of Anuradhapura, Sri Lanka.

## 2. Methods

A cross-sectional analytical study was conducted in government secondary schools in the Anuradhapura Municipal Council area of Sri Lanka to explore the epidemiology of allergic rhinitis among adolescents aged 13 to 14 years. Anuradhapura is a rural town located in the dry zone of Sri Lanka with surrounding paddy cultivations, farmlands, and forests. The study population consisted of 5,720 grade 8 and 9 students from 9 schools that are registered in the Education Department of North Central Province, situated within the Anuradhapura Municipal Council area. Schools and classes were selected through a multistage random sampling method until the required minimum sample size of 847 was recruited to the study. The minimum sample size was calculated considering the prevalence, precision, and anticipated dropout rate of 29.6% [19], 2%, and 10%, respectively. Administrative approval for conducting the study was obtained from the Provincial Department of Education of the North Central Province, the Zonal Education Office of Anuradhapura, and the principals of each school. Ethical clearance for the study was granted by the Ethics Review Committee of the Faculty of Medicine and Allied Sciences, Rajarata University of Sri Lanka (ERC/2022/42).

Information sheets, consent, and assent forms were distributed among parents or guardians and participants in their native languages of Sinhala and Tamil. Written informed consent was obtained from parent(s) or guardian(s), and assent was obtained from the participants. The prevalence and associated factors of allergic rhinitis were assessed using the translated and validated version of the International Study of Asthma and Allergy in Childhood (ISAAC) questionnaire (Supplementary Information 1 https://links.lww.com/PA9/A83) [20]. The questionnaire on the basic demographic data and risk factors was based on questionnaires used in previously published studies using the ISAAC methodology, and it was used to collect data on food and domestic environmental triggers and household risk factors. Allergic rhinitis was defined by a positive answer to whether the participant had nasal symptoms (sneezing, runny nose, or blocked nose) in the past year, unrelated to a cold or flu. Severe asthma was defined as the presence of at least 1 wheezing episode in the past 12 months, along with either 4 or more wheezing episodes, wheeze severe enough to affect speech, or sleep disturbance due to wheezing occurring at least once per week [20], and a case of eczema was identified when all 3 of the following were reported: a recurrent itchy rash for at least 6 months, presence of the rash in the past year, and involvement any of the following places: folds of the elbow, behind the knees, in front of the ankles, under the buttocks, or around the neck, ears or eyes. Information on demographic characteristics, symptomatology, housing conditions, exposure to allergens, physical activity levels, and dietary habits was collected. The study assessed the prevalence of allergic rhinitis and compared the number of adolescents with allergic rhinitis, eczema, and severe asthma.

Associated factors of allergic rhinitis were evaluated using the chi-square test, considering a *P* value less than 0.05 as statistically significant. The chi-square test was also performed after removing children with severe asthma and eczema to remove the confounding effect. A binary logistic regression analysis was performed, including variables showing a *P* value below 0.20 to identify independently associated factors of allergic rhinitis. Furthermore, multiple regression analysis using the backward conditional selection method was applied to assess the impact of environmental factors on the number of children with allergic rhinitis complaining of symptoms in a specific month. Environmental factors evaluated included the local standardized scores of highest, lowest, and average monthly temperatures; highest, lowest, and average monthly humidity; monthly rainfall; wind flow rate; and dew point. Subsequently, a predictive model for exacerbations of allergic rhinitis symptoms was developed using these environmental factors. The detailed epidemiology of severe asthma and eczema is published elsewhere [21, 22].

## 3. Results

A total of 1,029 participants were included in the study, comprising 528 (51.3%) males and 501 (48.7%) females. Most of the participants (n = 826, 80.3%) were aged 13 years, and 203 (19.7%) were aged 14 years. Allergic rhinitis symptoms were reported at least once in their lifetime by 387 (37.6%; 95% confidence interval [CI], 34.6–40.7), while ongoing allergic rhinitis, defined as experiencing symptoms within the past 12 months, was identified in 266 (25.9%; 95% CI, 23.3–28.6) adolescents. Itchy, watery eyes were reported alongside nasal symptoms in 142 (13.8%; 95% CI, 11.8–16.1) adolescents. Nasal symptoms interfered with activities of daily living in 195 (19.0%; 95% CI, 16.6–21.5), with moderate and severe disruption reported in 38 (3.7%; 95% CI, 2.6–5.0) and 16 (1.6%; 95% CI, 0.9–2.5), respectively.

**Figure 1.**
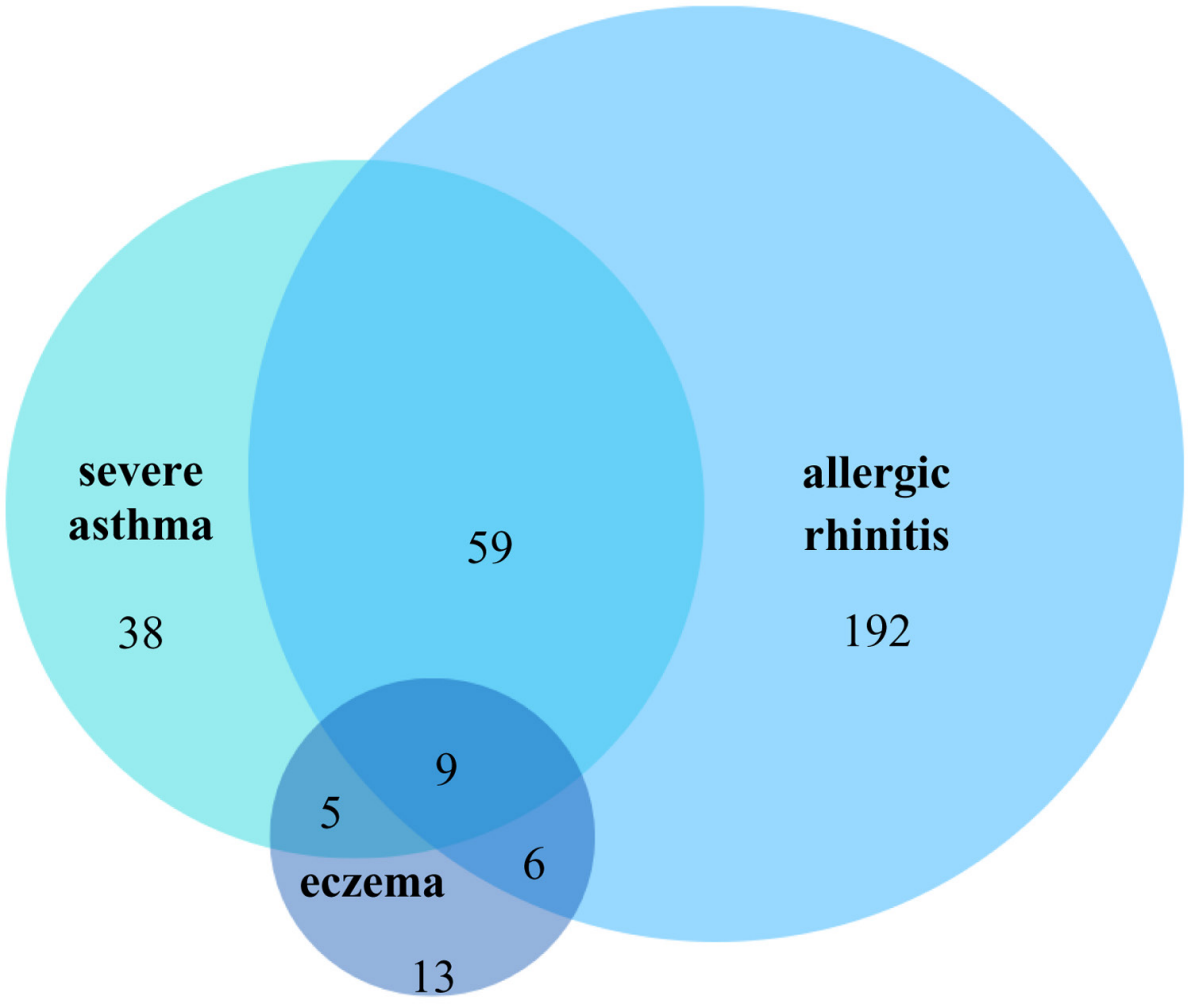
Association of allergic rhinitis, severe asthma, and eczema among 13 to 14-year-old adolescents in the Anuradhapura Municipal Council area, Sri Lanka (N = 1029). Figure 1 is a Venn diagram of adolescents with allergic rhinitis, severe asthma, and eczema. The number of adolescents with allergic rhinitis who also have severe asthma and eczema was 59 and 6, respectively. Severe asthma and eczema coexisted in 5 adolescents within the study sample. The number of adolescents with allergic rhinitis, severe asthma, and eczema was only 192, 38, and 13, respectively. Nine adolescents had all 3 diseases.

Severe asthma and eczema affected 157 (15.3%, 95% CI, 13.1–17.6) and 33 (3.2%, 95% CI, 2.1–4.3) adolescents, respectively, as previously published [20, 21]. Out of the 266 adolescents with allergic rhinitis, comorbid severe asthma and comorbid eczema were found in 68 (25.56%) and 15 (5.64%) adolescents, as opposed to only 43 (5.64%) and 18 (2.36%) detected in adolescents without allergic rhinitis (*P* < 0.01) (Fig. 1).

**Figure 2.**
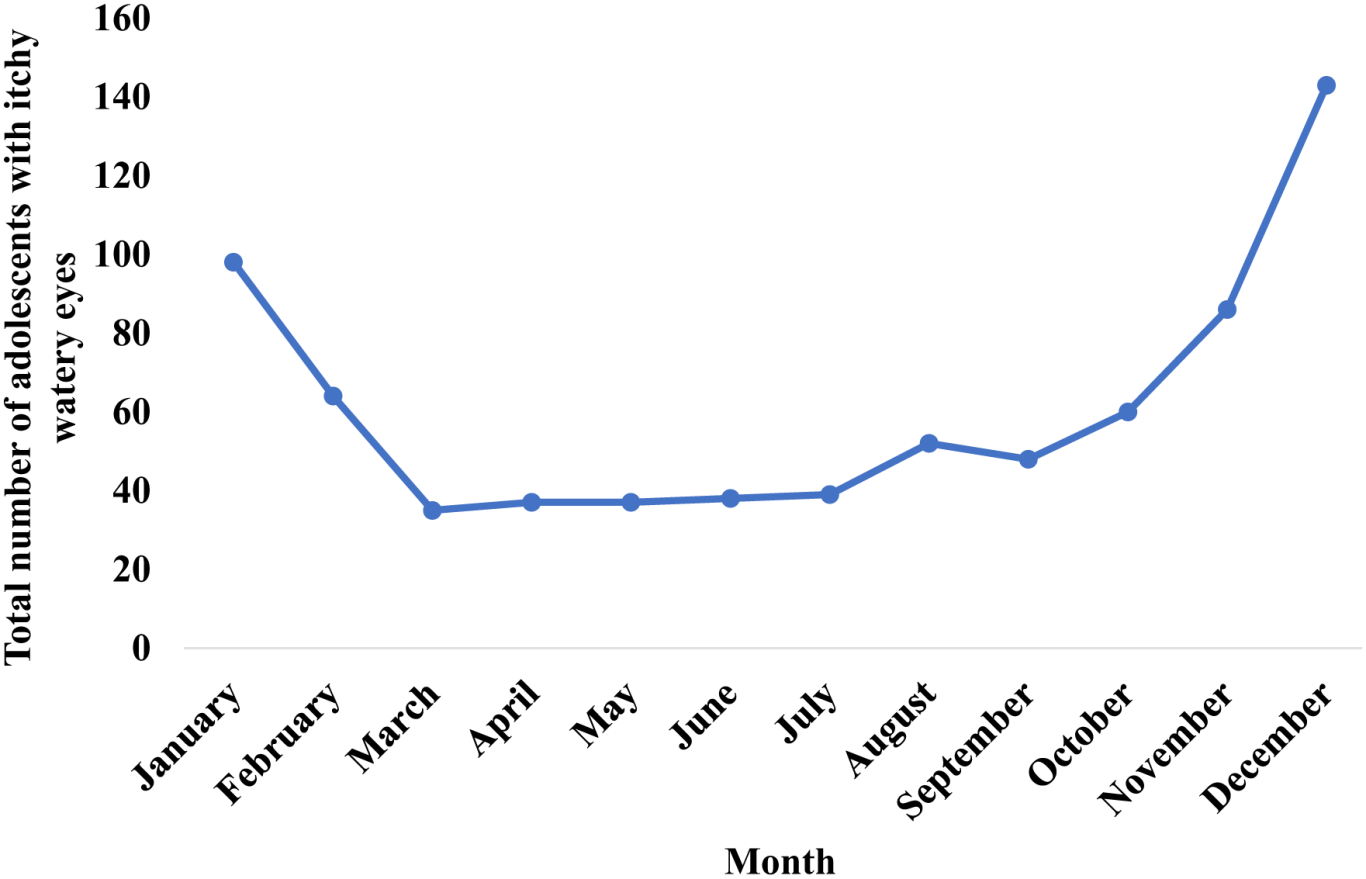
Temporal variation of itchy watery eyes among 13 to 14-year-old adolescents in the Anuradhapura Municipal Council area, Sri Lanka (N = 1029). Figure 2 shows the total number of adolescents with itchy, watery eyes each month from January to December. The total number in each month from January to December was 98, 64, 35, 37, 36, 38, 39, 52, 48, 60, 86, and 143. The highest numbers were reported from October to February.

Table 1 summarises the bivariate analysis of demographic characteristics, dietary habits, behavioral patterns, familial influences, and household factors associated with allergic rhinitis. Of these factors, severe asthma (*P* < 0.01), eczema (*P* = 0.01), engaging in play activities 4 to 6 times per week (*P* = 0.04), and sleeping on a rubber mattress (*P* = 0.05) were associated with allergic rhinitis. Engaging in play 4 to 6 times a week was significantly associated with allergic rhinitis even after removing adolescents with current asthma or eczema (*P* = 0.04) (Supplementary Information 2, https://links.lww.com/PA9/A84). Factors that were significant at a *P* value of 0.20 from the bivariate analysis presented in Table 1 were entered into the binary logistic regression analysis. In binary logistic regression, severe asthma (*P* < 0.01, adjusted odds ratio 5.75; 95% CI, 3.79–8.72) was independently associated with allergic rhinitis. Sleeping on a mattress made from a rubber-coir blend, and playing 4 to 6 times a week had adjusted odds ratios of 1.61 (*P* = 0.07; 95% CI, 0.95–2.74), and 1.50 (*P* = 0.06; 95% CI, 0.98–2.29), respectively, suggesting possible positive associations. Nasal symptoms occurred more frequently between October and February (Fig. 2). Backward selection in multiple regression analysis was employed to identify factors influencing the monthly number of children with allergic rhinitis presenting symptoms. The fifth step with the greatest statistical significance (F = 42.6, *P* < 0.001) was selected. The final model included 3 independent variables: standardized highest monthly temperature, standardized monthly precipitation, and standardized highest monthly humidity (Table 2). Details on all the variables included in multiple regression analysis are summarized in Supplementary Information 3 https://links.lww.com/PA9/A85. The total variability in temporal changes of allergic rhinitis predicted by the model (adjusted R-squared) was 94.1%. The model underwent validation to ensure there was no autocorrelation present (Durbin-Watson (DW) = 2.13). The standardized scores of the highest monthly temperatures (VIF 8.6, Tolerance 0.1) and humidity (Variance Inflation Factor (VIF) 8.0, Tolerance 0.1) showed significant multicollinearity. Therefore, we fitted a general linear model using the significant variables in the multiple regression to see the interaction effect of standardized scores of temperatures and humidity. The general linear model is significant (F = 33.17, *P* < 0.001), and the model did not show heteroskedasticity (F test *P* = 0.86). Further details regarding the model are presented in Table 3. Precipitation is the only significantly associated variable to predict the number of adolescents presenting with nasal symptoms each month. In addition, the highest monthly temperature showed an association at a *P* value of 0.086.

**Table 1.** Associated factors with allergic rhinitis among 13 to 14-year-old adolescents in the Anuradhapura Municipal Council area, Sri Lanka (N = 1,029)

Risk factor	Adolescents with allergic rhinitis		Adolescents without allergic rhinitis		*P* value (χ^2^ test)	Unadjusted odds ratio	95% confidence interval	
	N	%	N	%			Lower	Upper
Demographic factors								
Male gender	133	50.00	395	51.77	0.62	1.07	0.81	1.42
Born in Anuradhapura district	240	90.23	691	90.56	0.87	0.96	0.60	1.54
Housing conditions (having)								
Cement floor	154	57.89	426	55.83	0.56	1.09	0.82	1.44
Marble tile floor	107	40.23	333	43.64	0.33	0.87	0.65	1.15
Clay tile roof	44	16.54	162	21.23	0.12	0.75	0.52	1.08
Asbestos roof	199	74.81	553	72.48	0.29	1.20	0.86	1.67
Plastered walls	261	98.12	751	98.43	0.74	0.83	0.29	2.39
Exposure to allergens								
Having domestic dogs	176	66.17	521	68.28	0.53	0.91	0.68	1.22
Having domestic cats	109	40.98	277	36.30	0.18	1.22	0.92	1.62
Having domestic birds	43	16.17	129	16.91	0.78	0.95	0.65	1.38
Having close contact with an animal	145	54.51	413	54.13	0.91	1.02	0.77	1.34
Exposure to smokers at home	29	10.90	65	8.52	0.25	1.31	0.83	2.09
Frequent use of mosquito repellent coils	93	34.96	224	29.36	0.09	1.29	0.96	1.74
Frequent use of incense burners	177	66.54	487	63.83	0.43	1.13	0.84	1.51
Using LP gas for cooking	150	56.39	433	56.75	0.92	0.99	0.74	1.31
Using wood for cooking	88	33.08	241	31.59	0.65	1.07	0.80	1.44
Using electricity for cooking	33	12.41	101	13.24	0.73	0.93	0.61	1.41
Using sawdust for cooking	2	0.75	3	0.39	0.61	1.92	0.32	11.55
Sleeping on a rubber mattress	238	89.47	711	93.18	**0.05**	0.62	0.38	1.01
Sleeping on a rubber-mixed coir mattress	25	9.40	49	6.42	0.11	1.51	0.91	2.50
Physical activity^*^								
Play 4–6 times per week	42	15.79	84	11.01	**0.04**	1.52	1.02	2.26
Play 2–3 times a week	69	25.94	213	27.92	0.53	0.90	0.66	1.24
Play once a week	55	20.68	153	20.05	0.83	1.04	0.74	1.47
Play once a month	26	9.77	67	8.78	0.63	1.13	0.70	1.81
Dietary habits (Frequent consumption of)								
Pineapple^†^	21	7.89	42	5.50	0.16	1.47	0.85	2.53
Tomato^†^	78	29.32	214	28.05	0.69	1.06	0.78	1.45
Tuna fish^†^	84	31.58	225	29.49	0.52	1.10	0.82	1.49
King coconut^†^	34	12.78	92	12.06	0.76	1.07	0.70	1.63
Sour banana^†^	51	19.17	146	19.13	0.99	1.00	0.70	1.43
Ladies’ fingers^†^	65	24.44	161	21.10	0.26	1.21	0.87	1.68
Curd^†^	30	11.28	68	8.91	0.26	1.30	0.83	2.05
Milk powder^†^	83	31.20	232	30.41	0.78	1.04	0.77	1.41
Ridge gourd^†^	32	12.03	101	13.24	0.61	0.90	0.59	1.37
Centella^†^	67	25.19	199	26.08	0.77	0.95	0.69	1.32
Other disease conditions								
Comorbid severe asthma	68	25.56	43	5.64	**<0.01**	5.75	3.81	8.69
Comorbid eczema	15	5.64	18	2.36	**0.01**	2.47	1.23	4.98

* Physical activity was assessed with the question “How often do you play until sweating?”

† Consumption on more than 2 occasions per week is considered frequent.

Significance of bold values are *P* ≤ 0.05.

**Table 2.** Variables included in the multiple regression model for the total number of participants having allergic rhinitis symptoms in each month

Environmental factor (standardized)	Unstandardized coefficient	Standardized coefficient (beta)	T value	Significance	95% CI of unstandardized coefficient	
					Lower	Upper
Highest monthly temperature	−21.04	−6.42	−2.56	0.034	−40.01	−2.06
Monthly precipitation	30.19	0.92	6.48	0.000	19.45	40.93
Monthly humidity	−20.62	−0.63	−2.60	0.032	−38.93	−2.32
Intercept (c)	61.417		22.822	<0.001	55.211	67.622

**Table 3.** Parameter details of the general linear model for the total number of participants presenting with allergic rhinitis in each month

Parameter (standardized)	Coefficient	95% confidence interval		F	Significance (*P* value)
		Lower	Upper		
Highest monthly temperature	−17.475	−38.135	3.185	4.00	0.86
Humidity	−12.624	−37.858	12.611	1.399	0.275
Precipitation	26.159	12.283	40.035	19.872	0.003
Temperature-humidity interaction effect	−5.288	−16.617	6.040	1.218	0.306
Intercept (c)	56.892	45.342	68.441	135.676	<0.001

## 4. Discussion

This study explores the epidemiology of allergic rhinitis among adolescents living in Anuradhapura, Sri Lanka, revealing that over a quarter of the adolescents have current symptoms of allergic rhinitis, while over one-tenth experienced eye symptoms. Severe asthma was independently associated with allergic rhinitis while sleeping on a mattress made from a rubber-coir blend, and playing 4 to 6 times per week had higher odds for allergic rhinitis. Nasal symptoms were more common from October to February, with monthly precipitation significantly influencing the temporal changes in symptom occurrence.

The prevalence of allergic rhinitis ever, current allergic rhinitis, and rhinoconjunctivitis in the current study was 37.6%, 25.9%, and 13.8%, which is lower than the global prevalence of allergic rhinitis ever (42.1%, *P* < 0.01), current allergic rhinitis (33.2%, *P* < 0.01), and rhinoconjunctivitis (15.1%, *P* = 0.24) reported in the ISAAC study [12]. However, the overall prevalence of allergic rhinitis (29.3%, *P* < 0.01), current allergic rhinitis (23.4%, *P* = 0.06), and rhinoconjunctivitis (10.0%, *P* < 0.01) in the Indian subcontinent was less than that observed in the present study [12]. The national level prevalence of current allergic rhinitis was 29.6% (*P* = 0.02), and eye symptoms were 15.2% (*P* = 0.26) among the 13 to 14-year-old Sri Lankan adolescents, which was not significantly different from the current study [16]. A study conducted among rural preschool children in the Anuradhapura district reported the prevalence of allergic rhinitis as 11.6% (95% CI, 9.7–13.5), which was significantly lower than the current study (*P* < 0.01) [23]. The higher prevalence of allergic rhinitis among adolescents follows the expected pattern of increasing incidence till adolescence [10]. This pattern is probably attributable to the gradual sensitization to airborne, dietary, and other allergens that contribute to the development and expression of atopic disease later in life [24].

Severe asthma was independently associated with allergic rhinitis in the current study. Asthma is a well-known common comorbidity in patients with allergic rhinitis, with studies reporting that up to 38% of individuals with allergic rhinitis also experience asthma [6, 25]. These 2 conditions share common genetic predispositions and environmental triggers, indicating shared underlying mechanisms and inflammatory processes [25, 26]. They are often considered manifestations of a single syndrome known as chronic allergic respiratory syndrome [26, 27]. The impact of rhinitis on the nasal passages can extend to the lower airways, exacerbating asthma symptoms [26]. This association suggests screening, early identification and intervention, integrated care, and trigger avoidance in the prevention and management of both conditions. Both sleeping on a rubber-mixed-coir mattress and playing 4 to 6 times per week had higher odds of allergic rhinitis, suggesting potential associations that warrant further exploration. Sleeping on a rubber-mixed coir mattress has been suggested as a potentially significant source of exposure to house dust mites and mold because rubber-coir mattresses are manufactured using coconut fibers treated with latex solutions [28, 29]. There is a significant correlation between the severity of nasal itching and high levels of exposure to house dust mite allergens, as observed in both skin prick tests and specific immunoglobulin E test results [28, 30]. Therefore, sleeping on a rubber-mixed coir mattress is a preventable risk factor that should be avoided in the local context. Previous studies conducted on 13 to 14-year-old adolescents have also reported that a positive association exists between frequent physical activity and current symptoms of allergic rhinitis [31]. Engaging in physical activity outdoors may potentially increase exposure to outdoor allergens, including pollens and other airborne particles, worsening allergic rhinitis symptoms [32]. Given that sleeping on a rubber-mixed coir mattress and physical activity are both linked to allergic rhinitis in the context of allergen exposure, indoor allergens tend to persist throughout the year, while outdoor allergens rise and fall according to periods of the year [33]. In the local context, despite poor outdoor air quality, particularly in urban areas, literature reveals that poor indoor air quality contributes more towards respiratory problems [34].

Nasal symptoms were more prominent in the months from October to February. The country, located near the equator, has a tropical climate characterized by hot and humid weather all year round. The widespread rainfall from October to mid-December is known as the second inter-monsoon period. Subsequently, from mid-December to February, the northeast monsoon season brings relatively cooler temperatures and occasional cool nights. The current study shows that during monthly precipitation from October to February, adolescents with allergic rhinitis experience more exacerbations of nasal symptoms, which may be attributed to several factors. When it rains while grass and weed pollen levels are high, the raindrops can break up the clumps of pollen into smaller particles, which quickly disperse into the air. Rainy seasons often result in people spending more time indoors, leading to high exposure to indoor allergens, causing worsening of allergic rhinitis symptoms [35]. Furthermore, during periods of heavy rainfall, the common practice of keeping doors and windows closed for a longer duration may result in diminished natural ventilation and restricted airflow, reducing the effective exchange of indoor and outdoor air, thereby contributing to the accumulation of indoor air pollutants [36]. Moreover, the rainy season is associated with a higher incidence of viral rhinitis, which can mimic the symptoms of allergic rhinitis [37]. Nasal symptoms are identified more commonly in January and August to October in a previous study conducted in the same geographical area, among preschool children, which concurs with the association with the monsoonal rain periods [23].

## 5. Limitations

This cross-sectional study focused only on the associations between the factors and allergic rhinitis. Future studies are needed to explore the causal link between these factors and the development of allergic rhinitis. Information on the timing, duration, and magnitude of exposure to risk factors was not captured in this study. In Sri Lanka, it is commonly believed by the public, especially in rural populations, that certain food items worsen asthma and allergic rhinitis. However, such associations and their causality for symptom exacerbation remain unconfirmed [23]. The effect of portion size, intervals between consumption, long-term dietary habits, and other food-related factors was not assessed in the current study. Despite commonly held beliefs that food-related factors exacerbate allergic rhinitis, this study did not demonstrate the association between symptoms and dietary habits. The study did not assess the participants’ diagnosis and current management of allergic rhinitis. The current study suggests symptom exacerbation follows a temporal pattern predicted by monthly precipitation, but the air pollution, indoor allergen measurements, or viral infections could be confounders that are not addressed in the study.

## 6. Conclusions

This study showed that more than 1 in 4 adolescents have symptoms of current allergic rhinitis, while over one-tenth experienced eye symptoms. Severe asthma was independently associated with allergic rhinitis. Nasal symptoms were more common from October to February, with monthly precipitation playing a significant role in the variation of symptoms.

## Acknowledgments

The authors would like to acknowledge Dr. Kirthi Gunasekera, Consultant Chest Physician and National Coordinator for the Sri Lankan arm of the ISAAC study group, for granting permission to use the translated questionnaires. Additionally, the authors acknowledge the administrative permission and support provided by the Provincial Director of Education for the North Central Province, the Zonal Director of Education, and the principals and teachers of the schools located in the Anuradhapura Municipal Council area. The author, Shashanka Rajapakse, expresses their appreciation for the support from the International Cooperation & Education Program (NCCRI·NCCI 52210-52211, 2024) of the National Cancer Center, Korea.

This research project received no specific grant from any funding agency in the public, commercial or not-for-profit sectors.

## Conflicts of interest

The authors have no financial conflicts of interest.

## Author contributions

TS contributed to conceptualization, data curation, data visualization, formal analysis, investigation, methodology, project administration, and writing of the original draft. TCS, SS, VS, PSS, and ST contributed to conceptualization, data curation, data visualization, formal analysis, investigation, methodology, and writing of the original draft. LW contributed to data visualization, formal analysis, and writing the original draft. JW contributed with data curation, formal analysis, methodology, and writing – review and editing. SR contributed in conceptualization, data curation, data visualization, formal analysis, methodology, project administration, supervision, and writing – review and editing.

## Supplementary Material






