# Supplementary material for: Prevalence, associated factors, and temporal variation of allergic rhinitis among 13 to 14-year-old adolescents from rural Sri Lanka: An analytical cross-sectional study
Source: Asia Pac Allergy. 2026 Jan 13;16(3):145–51. doi: 10.5415/apallergy.0000000000000252 (PMC13193263; doi:10.5415/apallergy.0000000000000252)
Supplement: Supplementary file 1 [file pa9-16-145-s001.pdf]

Risk factor questionnaire

1. You are a Boy ☐ Girl ☐
2. Your age is Years …………
3. Were you born in Sri Lanka? Yes ☐ No ☐
4.
   a. If yes in which district
      ……………………………………………
   b. If not in which country
      ……………………………………………
5. Do you have elder sisters or brothers? Yes ☐ No ☐
   a. If yes number of elder sisters
      ……………………………………………
   b. If yes number of elder brothers
      ……………………………………………
6. Floor of the room that you are sleeping is made of
   - Cement ☐
   - Clay ☐
   - Sand ☐
7. How many sleep in your room except you? ……………………………………………
8. Roof of your house is made of ?
   - Clay tiles ☐
   - Asbestos ☐
   - Soiled ☐
   - Cadjan ☐
   - Straw ☐
   - Other (name)
     ……………………….
9. Walls of your house is made of?
   - Plaster ☐
   - Clay/wattle ☐

Cadjan ☐
Timber ☐
Other (name) ………………………

10. Which type of animal(s) live in your home or garden?

`

Dogs ☐
Cats ☐
Birds/poultry ☐
Goats ☐
Cows/buffalos ☐
Others (name) ………………

11. Do you have a close relationship with those animals? Yes ☐ No ☐
12. Does anybody smoke in your household? Yes ☐ No ☐
13. Are the following used frequently in your house?

Mosquito coils ☐
Kerosene lamp ☐
Fragrance sticks ☐

14. What is the fuel used in your house for cooking?

Electricity ☐
Gas ☐
Wood/saw dust ☐
Others (name) ………………

15. Where do you sleep in

In bed ☐
On the floor ☐

16. What do you use for sleeping in

Rubber mattress ☐
Rubber mixed coir ☐

A clothe ☐

A mat ☐

17. How often do you play until sweating?

Everyday ☐

4-6 times a week ☐

2-3 times a week ☐

Once a week ☐

Once a month ☐

18. How often following food items/drinks are used?

Put a ✓ in the correct box

Food item/drink	Never	Seldom	Once a week	1-2 times a week	3-6 times a week	Once a day or frequently
pine apple						
Tomatoes						
Tuna fish						
King coconut water						
Sour banana						
Ladies fingers						
Curd						
Powdered milk						
Ridged gourd (jegfld¿)						
centella						

19. Do you feel sick/uncomfortable during Full moon Poya days?

Yes ☐ No ☐

20. If ‘yes’ write what it is.

…………………………………………………………………………………………

…………………………………………………………………………………………

…………………………………………………………………………………………

Core questionnaire for asthma

1. Have you <u>ever had</u> wheezing or whistling in the chest at any time in the past?

   Yes ☐ No ☐

   If your answer is "No" please move to question 6

---

2. Have you had wheezing or whistling in the chest <u>in the past 12 months?</u>

   Yes ☐ No ☐

   If your answer is "No" please move to question 6

---

3. How many attacks of wheezing have you had <u>in the past 12 months?</u>

   None ☐
   1-3 ☐
   4-12 ☐
   More than 12 ☐

---

4. <u>In the past 12 months</u> how often, on average, have your sleep been disturbed due to wheezing?

   Sleeping is disturbed due to wheezing attacks,

   Never ☐
   Yes, but not in every week ☐
   Yes, once or more than once a week ☐

5. In the past 12 months, has wheezing ever been severe enough to limit your speech to only one or two words at a time between breaths?

Yes ☐ No ☐

---

6. Have you ever had asthma? Yes ☐ No ☐
7. In the past 12 months has your child's chest sounded wheezy when/after playing?

   Yes ☐ No ☐

8. In the past 12 months, have you had a dry cough at night, apart from a cough associated with a cold or chest infection?

   Yes ☐ No ☐

Core questionnaire for rhinitis

1. Have you ever had a problem with sneezing or runny nose or blocked nose when you DID NOT have a cold or a flu?

   Yes ☐ No ☐

If your answer is "No" please move to question 6

---

2. In the past 12 months have you had a problem with sneezing or runny nose or blocked nose when you DID NOT have a cold or a flu?

   Yes ☐ No ☐

If your answer is "No" please move to question 6

---

3. In the past 12 months has this nose problem been accompanied by itchy watery eyes?

   Yes ☐ No ☐

4. In which of the past 12 months did this nose problem occur?

   January ☐ May ☐ September ☐

   February ☐ June ☐ October ☐

March ☐ July ☐ November ☐

April ☐ August ☐ December ☐

5. In the past 12 months how much did this nose problem interfere with your daily activities?

Not at all ☐

A little ☐

A moderate amount ☐

A lot ☐

6. Have you ever infected with rhinitis limited to some months of the year?

Yes ☐ No ☐

Core questionnaire for eczema

1. Have you ever had an itchy rash which was periodical at least for 6 months?

Yes ☐ No ☐

If your answer is "No" please move to question 7

---

2. Have you had this itchy rash at any time in the past 12 months?

Yes ☐ No ☐

If your answer is "No" please move to question 7

---

3. Has this itchy rash at any time affected any of the following places?
In the folds of the elbow, behind the knees, in front of the ankles, under the buttocks, or around the neck, ears or eyes?

Yes ☐ No ☐

4. At what age the itchy rash first appears?

Before 2 years old ☐

2-4 years ☐

5 years old more ☐

5. Has this rash cleared completely at any time during the <u>past 12 months?</u>

Yes ☐ No ☐

6. In the past 12 months, how often, on average, have you been kept awake at night by this itch rash?

Never ☐

Yes but not in every week ☐

Yes, once or more in a week ☐

7. Have you had itchy rash (eczema) on skin? Yes ☐ No ☐

හේතු සාධක සඳහා ප්‍රශ්ණාවලිය

1 ඔබ ගැහැණු පිරිමි

2 ඔබේ වයස කීයද ? අවුරුදු

3 ඔබ ඉපදුනේ ශ්‍රී ලංකාවේද? ඔව් නැත

4 **A** ඔව් නම් කුමන දිස්ත්‍රික්කයේද ? ……………………………………..

4 **B** නැත්නම් කුමන රටේදැයි ලියන්න ……………………………………..

5 ඔබට වඩා වැඩිමහල් සහෝදර සහෝදරියන් සිටීද? ඔව් නැත

ඔව් නම් වැඩිහිටි සහෝදරියන් ගණන

ඔව් නම් වැඩිහිටි සහෝදරයන් ගණන

7 ඔබ හැර එම කාමරයේ තව කී දෙනෙකු නිදාගන්නවාද ?

8 ඔබ නිදන කාමරයේ පොළව සාදා ඇත්තේ

සිමෙන්ති

මැටි

වැලි

වෙනත් ද්‍රව්‍ය (නම් කරන්න)..

9 ඔබේ ගෙදර වහලය සාදා ඇත්තේ

උළු

ඇස්බැස්ටෝස්

ටකරන්

පොල් අතු

පිදුරු

වෙනත් ද්‍රව්‍ය (නම් කරන්න)..

10 ඔබේ ගෙදර බිත්ති කුමන වර්ගයේද ?

කපරාරු කරන ලද

මැටි / වරිච්චි

පොල් අතු

ලෑලි

වෙනත් ද්‍රව්‍ය(නම් කරන්න)……………………………………………………………………..

11 ඔබේ ගෙදර හෝ ගෙවත්තේ කුමන වර්ගයේ සතුන් සිටීද ?

බල්ලන් ☐

බළලුන් ☐

කුරුල්ලන්/කුකුළන් ☐

එළුවන් ☐

හරක්/මී හරක් ☐

වෙනත් (නම් කරන්න)..

12 ඔබට එම සතුන් සමග සමීප ඇසුරක් තියනවාද ?

ඔව් ☐

නැත ☐

14 දැනට ඔබේ නිවසේ දුම් පානය කරන්නන් සිටිනවාද ?

ඔව් ☐

නැත ☐

15 පහත සඳහන් ද්‍රව්‍ය ඔබේ නිවසේ නිතර පාවිච්චි කරනවාද ?

මදුරු දඟර ☐

කුප්පි ලාම්පු ☐

හඳුන් කූරු ☐

16 ඔබේ ගෙදර කෑම පිසීම සඳහා පාවිච්චි කරන්නේ මොනවාද ?

විදුලිය ☐

ගෑස් ☐

දර/ ලී කුඩු ☐

වෙනත් ද්‍රව්‍ය (නම් කරන්න)..

17 ගෙදර ඔබ නිදා ගන්නේ

ඇඳක

බිම ☐

18 ඔබ නිදා ගැනීමට පාවිච්චි කරන්නේ ☐

රබර් මෙට්ටයක් ☐

රබර් මිශ්‍රිත කොහු මෙට්ටයක් ☐

රෙද්දක් ☐

පැදුරක් ☐

19 දහඩිය දමා මහන්සි වෙන තුරු ඔබ සෙල්ලම් කරන්නේ

සෑමදාම ☐

සතියකට 4-6 වතවක් ☐

සතියකට 2-3 වතාවක් ☐

සතියකට වරක් ☐

මාසයකට වරක් ☐

20 සාමාන්‍යයෙන් පහත සඳහන් දේ කෑමට හෝ බීමට ගන්නේ

නිවැරදි ✓ කොටුවේ ලකුණ යොදන්න

ආහාරය	නැත	ඉඳහිට	සතියට වරක්	සතියට 1-2	සතියට 3-6	දවසකට වරක් හෝ නිතර
අන්නාසි						
තක්කාලි						
බලයා/ කෙලවල්ලා මාලු						
තැඹිලි වතුර						
ඇඹුල් කෙසෙල්						
බණ්ඩක්කා						
මුදවපු කිරි						
කිරිපිටි වර්ග						
වැටකොළු						
ගොටුකොළ						

21 ඔබට පෝය දවස් වලට ඇඟට වෙනසක් හෝ අසනීප ගතියක් දැනෙනවාද ?

ඔව් ☐

නැත ☐

22 එසේ නම් එය කුමක්දැයි මෙහි ලියන්න

....

....

ඇදුම සඳහා ප්‍රශ්නාවලිය

1. ඔබට මීට පෙර <u>කිසියම් දිනක හතිය</u>(පපුවේ මහන්සිය) හෝ පපුවේ රාං රූං ගාන ශබ්දයක් ඇති වී තිබේද? — ඔව් ☐ / නැත ☐

ඔබේ පිළිතුර 'නැත' නම්කරුණාකර 6 වන ප්‍රශ්නයට යන්න.

---

2. ඔබට <u>පසුගිය මාස 12 ඇතුලත</u> හතිය(පපුවේ මහන්සිය) හෝ පපුවේ රං රූං ශබ්දයක් තිබුණාද? — ඔව් ☐ / නැත ☐

ඔබේ පිළිතුර 'නැත' නම් කරුණාකර 6 වන ප්‍රශ්නයට යන්න.

---

3. ඔබට <u>පසුගිය මාස 12 ඇතුලත</u> හතිය (පපුවේ මහන්සිය) කීවරක් ඇතිවී තිබේද?

- නැත ☐
- 1-3 ☐
- 4-12 ☐
- 12 ට වැඩි ☐

---

4. <u>පසුගිය මාස 12 ඇතුලත,</u>සාමාන්‍ය වශයෙන් කීවරක් හතිය (පපුවේ මහන්සිය) නිසා ඔබගේ, නින්දට බාධා ඇතිවුනාද?

හතිය (පපුවේ මහන්සිය) නිසා කිසිදිනක නින්දෙන් අවදි වී

- නැත ☐
- ඇත නමුත් සෑම සතියකම නොවේ ☐
- ඇත සතියකට වරක් හෝ ඊට වැඩි වාරණනක්. ☐

5. ඔබට <u>පසුගිය මාස 12 ඇතුලත</u> එක හුස්මකදී වචන 2ක් වත් කථා කිරීමට බැරි තරම් තදින් හතිය (පපුවේ මහන්සිය) තිබුනාද? — ඔව් ☐ / නැත ☐

---

6. ඔබට <u>කවදා හෝ</u> අදුම සෑදී ඇත්ද? — ඔව් ☐ / නැත ☐

7. ඔබට <u>පසුගිය මාස 12 ඇතුලත</u> සෙල්ලම් කරන විට හෝ ඉන් පසු පපුවේ මහන්සි ශබ්දයක් හැදුනාද? — ඔව් ☐ / නැත ☐

8. ඔබට <u>පසුගිය මාස 12</u> ඇතුලත, සෙම්ප්‍රතිශ්‍යාව හෝ පෙනහලු ආසාදනයක් සමග ඇති වු කැස්ස හැරුණුවිට, රාත්‍රියට වියලි කැස්සක් ඇතිවුනාද? — ඔව් ☐ / නැත ☐

ඇදුම ආශ්‍රිත ආසාත්මිකතාවය (පීනස) සඳහා ප්‍රශනාවලිය

1. ඔබට උණ සෙම්ප්‍රතිශ්‍යාව, හෝ සෙම්ප්‍රතිශ්‍යාවවැලදී <u>නොමැති</u> අවස්ථාවක කිවිසුම් යාම,නාසයෙන් දියර ගැලීම හෝ නාසයේතදවීමක් නිසා,<u>කිසියම් දිනක</u>, අමාරුවක් ඇති වී තිබේද?

ඔව් ☐
නැත ☐

ඔබේ පිළිතුර 'නැත' නම් කරුණාකර 6 වන ප්‍රශ්නයට යන්න.

---

2. ඔබට <u>පසුගිය මාස 12</u> ඇතුළත සෙම්ප්‍රතිශ්‍යාවහෝඋණ සෙම්ප්‍රතිශ්‍යාව හෝ වැලදී නොමැති අවස්ථාවක,කිවිසුම් යාම හෝ නාසයෙන් දියර ගැලීමහෝ නාසයේ තදවීමක් හේතුවෙන් අමාරුවක් ඇතිවී තිබේද?

ඔව් ☐
නැත ☐

ඔබේ පිළිතුර 'නැත' නම් කරුණාකර 6 වන ප්‍රශ්නයට යන්න.

---

3. ඔබට <u>පසුගිය මාස 12 ඇතුළත</u>, නාසයේ ඇතිවූ අමාරුවත් සමග ඇස්වල කැසීමක් කඳුළු ගැලීමක් සිදුවූවාද?

ඔව් ☐
නැත ☐

4. මේ නාසයේ අමාරුව ඇතිවුයේ <u>පසුගිය මාස 12 න්</u> කිනම් මාසවලද?

කරුණාකර අදල මාසවලට 'හරි' (✓) ලකුණ යොදන්න.

ජනවාරි,	☐	මැයි,	☐	සැප්තැම්බර්,	☐
පෙබරවාරි,	☐	ජුනි,	☐	ඔක්තෝම්බර්,	☐
මාර්තු,	☐	ජුලි,	☐	නොවැම්බර්,	☐
අප්‍රේල්,	☐	අගෝස්තු,	☐	දෙසැම්බර්,	☐

5. ඔබට <u>පසුගිය මාස 12 ඇතුළත</u>, නාසයේ අමාරුව නිසා, එදිනෙදා කටයුතු වලට කෙතරම් බාධා පැමිණියේද?

ඇත්තෙන්ම නැහැ ☐
සුළු වශයෙන් ☐
මධ්‍යස්ථ වශයෙන් ☐
ගොඩක් ☐

6. ඔබට <u>කවදා හෝ</u> අවුරුද්දේ සමහර මාස වලට පමණක් සීමා වු පීනස වැළඳීතිබුනාද?

ඔව් ☐
නැත ☐

දදය (එක්සීමාව) සඳහා මූලික ප්‍රශ්නාවලිය

1. ඔබට <u>කවදා හෝ</u> අඩු වශයෙන් මාස 6ක් වත් වීටින්විට පැවතුන කසන දදයක් තිබුණාද? ඔව් ☐ නැත ☐

පිළිතුර 'නැත' නම් කරුණාකර 7 වන ප්‍රශ්නයට යන්න.

2. ඔබට පසුගිය <u>මාස 12 ඇතුළත</u>, කිසියම් දිනක මෙම කසන දදය සෑදී තිබේද? ඔව් ☐ නැත ☐

පිළිතුර 'නැත' නම් කරුණාකර 7 වන ප්‍රශ්නයට යන්න.

3. මෙම කසන දදය.<u>කවදා හෝ</u> පහත සඳහන් කර ඇති එක ස්ථානයකවත් ඇතිවී තිබේද?

වැලමිටේ නැමෙනතැන්වල, දනහිස පිටුපස, වළලුකර ඉදිරිපස, තට්ටම යට,ගෙලඇස් හෝ කන් අවට ඔව් ☐ නැත ☐

4. මෙම කසන දදය පලමුවෙන්ම ඇතිවුයේ කුමන වයසේදීද?

අවු.2 ට අඩු ☐

අවු.2.4 ☐

අව.5 හෝ ඊට වැඩි ☐

5. <u>පසුගිය මාස 12 ඇතුළත</u>, කිසියම් කාලයක මෙම දදය සම්පූර්ණයෙන්ම නැති වී ගියේද? ඔව් ☐ නැත ☐

6. ඔබ <u>පසුගිය මාස 12 ඇතුළත</u>, මෙම කසන දදය නිසා, සාමාන්‍ය වශයෙන් කීවරක්, රාත්‍රි කාලයේ අව්දි වී සිටියාද?

පසුගිය මාස 12 ඇතුළත කවදාවත් නැත. ☐

ඇත නමුත් සෑම සතියකම නොවේ ☐

ඇත සතියකට වරක් හෝ ඊට වැඩි වාරගාණක් ☐

7. ඔබට කවදා හෝ සමේ කසන දදයක් (එක්සීමාවක්/රක්තයා)තිබුණාද? ඔව් ☐ නැත ☐

ஆபத்தேற்படுத்தும் காரணிகள் தொடர்பான கேள்விகள்

1. பாலினம் பெண்/ஆண்

2. வயது வருடங்கள் ………………

3. நீங்கள் இலங்கையை பிறப்பிடமாக கொண்டவரா? ஆம்/இல்லை

4.
   a. ஆம் எனின் பிறந்த மாவட்டம் ……………………………………………
   b. இல்லை எனின் எந்த நாடு ……………………………………………

5. உங்களுக்கு மூத்த சகோதர சகோதரிகள் உள்ளனரா? ஆம்/இல்லை
   a. ஆம் எனின் மூத்த சகோதரிகளின் எண்ணிக்கை ……………………………………………
   b. ஆம் எனின் மூத்த சகோதரர்களின் எண்ணிக்கை ……………………………………………

6. நீங்கள் உறங்கும் அறையின் தரை எதனால் ஆக்கப்பட்டது?
   - சீமெந்து ☐
   - களி ☐
   - மணல் ☐

7. உங்களைத் தவிர உங்கள் அறையில் உறங்கும் ஏனையவர்களின் எண்ணிக்கை?
   ……………………

8. உங்களது வீட்டின் கூரை எதனால் ஆக்கப்பட்டுள்ளது?
   - டைல் ☐
   - அஸ்பஸ்டோஸ் ☐
   - soiled ☐
   - Cadjan ☐
   - வைக்கோல் ☐
   - வேறு (பெயர்) ………………………..

9. உங்களது வீட்டின் சுவர் எதனால் ஆக்கப்பட்டுள்ளது?
   - Plaster ☐
   - களி 2 ☐
   - Cadjan ☐
   - புலகை ☐
   - வேறு (பெயர்) …………………………

10. வீட்டில் தோட்டத்தில் வாழும் பிராணிகள்?
    - நாய் ☐
    - பூனை ☐
    - புறவைகள், கோழி ☐
    - ஆடு ☐
    - மாடு, எருமை ☐
    - வேறு (பெயர்) ………………………

11. நீங்கள் அப்பிராணிகளுடன் நெருங்கி உறவாடுபவரா? ஆம்/இல்லை

12. உங்கள் அயற்சூழலில் புகைப்பவர்களின் நடமாட்டம் உண்டா? ஆம்/இல்லை

13. பின்வருவனவற்றை அடிக்கடி உபயோகிக்கின்றீர்களா?

நுளம்புச்சுருள் ☐
மண்ணெண்ணெய் விளக்கு ☐
வாசனை தனற்குச்சி ☐

14. உங்கள் வீட்டில் சமையலுக்கு பயன்படுத்தப்படும் எரிபொருள் யாது?

மின்சாரம் ☐
வாயு ☐
விறகு ☐
வேறு (பெயர்) ....

15. நீங்கள் உறங்குவது

கட்டிலில் ☐
தரையில் ☐

16. நீங்கள் உறங்க பயன்படுத்துவது

இறப்பர் மெத்தை ☐
இறப்பர் கலந்த தும்பு மெத்தை ☐
துணி ☐
பாய் ☐

17. நீங்கள் எவ்வெப்போது வியர்க்கும் வரை விளையாடுவீர்கள்?

தினமும் ☐
கிழமைக்கு 4-6 முறை வரை ☐
கிழமைக்கு 2-3 முறை வரை ☐
கிழமைக்கு ஒரு தடவை ☐
மாதத்துக்கு ஒரு தடைவ ☐

18. பின்வரும் உணவுப்பதார்த்தங்களை பயன்படுத்துவதன் அடிப்படையில் அட்டவனையில் ✓ அடையாளத்தை இட்டு நிரப்புக?

உணவு பானம்	ஒருபோதும் இல்லை	சிலபோது	கிழமைக்கு ஒரு முறை	கிழமைக்கு 1-2 முறை	கிழமைக்கு 3-6 முறை	தினமும், அடிக்கடி
அன்னாசி						
தக்காளி						
சூரை மீன்						
இளநீர்						
புளி வாழை						
வெண்டிக்காய்						
தயிர்						
பால் மா						
பீர்க்கு						
வல்லாரை						

19. பௌர்ணமி தினங்களில் உங்கள் குழந்தை நோய்வாய்படுகின்றாரா அல்லது ஏதேனும் அசௌகரியங்களுக்கு உள்ளாகின்றாரா?

ஆம் ☐
இல்லை ☐

20. ஆம் எனின் எவ்வாறு,

....
....
....

ஆஸ்த்துமா தொடர்பான வினாக்கள்

13-14 வயதினருக்கான வினாக்கள்

1. கடந்த காலத்தில் உங்களுக்கு இளைப்பு அல்லது மூச்சு விடும் போது நெஞ்சில் கீச்சிடும் சத்தம் இருந்ததா?

ஆம் ☐
இல்லை ☐

நீங்கள் "இல்லை" என்று விடையளித்திருப்பின் 6ம் வினாவிற்குச் செல்லவும்

2. கடந்த 12 மாதகாலத்தில் உங்களுக்கு இளைப்பு அல்லது மூச்சு விடும் போது நெஞ்சில் கீச்சிடும் சத்தம் இருந்ததா ?

ஆம் ☐
இல்லை ☐

நீங்கள் "இல்லை" என்று விடையளித்திருப்பின் 6ம் வினாவிற்குச் செல்லவும்

3. கடந்த 12 மாதகாலங்களில் நீங்கள் எத்தனை தடவை இளைப்பினால் பாதிக்கப்பட்டீர்கள்?

ஒரு போதும் இல்லை ☐
1 முதல் 3 முறை ☐
4 முதல் 12 முறை ☐
12 முறைக்கு மேல் ☐

4. கடந்த 12 மாதகாலங்களில் பொதுவாக எத்தனை தடவை இளைப்புக் காரணமாக உங்களது தூக்கம் குழம்பியுள்ளது?

நான் தூக்கத்தால் ஒரு போதும் எழும்பவில்லை ☐
எழும்பியுள்ளேன் ஆனால் ஒவ்வொரு கிழமையும் இல்லை ☐
கிழமையில் ஒன்று அல்லது அதற்கு மேற்பட்ட இரவுகள் எழும்பியுள்ளேன் ☐

5. <u>கடந்த 12 மாதகாலங்களில்</u> கடுமையான இளைப்புக் காரணமாக உங்களுக்கு ஒரே மூச்சில் ஒன்று அல்லது இரண்டு சொற்களுக்கு மேல் பேச முடியாமல் இருந்ததா?

ஆம் ☐
இல்லை ☐

---

6. உங்களுக்கு ஆஸ்துமா <u>எப்போதாவது</u> வந்ததுண்டா?

ஆம் ☐
இல்லை ☐

7. <u>கடந்த 12 மாதகாலங்களில்</u> உங்களுக்கு உடற்பயிற்சி விளையாடும் போது அல்லது அதன் பின்பு நெஞ்சில் கீச்சிடும் சத்தம் ஏற்பட்டதா?

ஆம் ☐
இல்லை ☐

8. <u>கடந்த 12 மாதகாலங்களில்</u> உங்களுக்கு எப்போதாவது நெஞ்சு தொற்று நோய்/தடிமன்னுடன் இல்லாமல் இரவில் வரண்ட இருமல் ஏற்பட்டதா?

ஆம் ☐
இல்லை ☐

ரினிடிஸ்(நாசியழற்சி) தொடர்பான கேள்விகள்

1. உங்கள் குழந்தை, தடுமல் அல்லது காய்ச்சல் இல்லாத சந்தர்ப்பங்களில் தும்மல் அல்லது மூக்கு ஒழுகுதல் அல்லது மூக்கடைப்பு என்பவற்றால் எப்போதாவது அவதியுற்றுள்ளாரா?

ஆம் ☐
இல்லை ☐

இல்லையாயின் வினா 6 இற்கு செல்க

2. கடந்த 12 மாதங்களில் உங்கள் குழந்தை, தடுமல் அல்லது காய்ச்சல் இல்லாத சந்தர்ப்பங்களில் தும்மல் அல்லது மூக்கு ஒழுகுதல் அல்லது மூக்கடைப்பு என்பவற்றால் எப்போதாவது அவதியுற்றுள்ளாரா?

ஆம் ☐
இல்லை ☐

இல்லையாயின் வினா 6 இற்கு செல்க

3. கடந்த 12 மாதங்களில் மூக்குடன் தொடர்பான அவதியடன், அரிப்புடன் நீர் வடியும் கண்கள் காணப்பட்டதா?

ஆம் ☐
இல்லை ☐

4. கடந்த எந்த 12 மாதங்களில் மூக்குடன் தொடர்பான இவ்அவதி ஏற்பட்டது?

ஜனவரி	☐	பெப்ரவரி	☐	மார்ச்	☐
ஏப்ரல்	☐	ஜுன்	☐	ஜுலை	☐
ஆகஸ்ட்	☐	செப்டெம்பர்	☐	ஒக்டோபர்	☐
நவம்பர்	☐	டிசம்பர்	☐		

5. கடந்த 12 மாதங்களில் மூக்குடன் தொடர்பான இவ்அவதியானது குழந்தையின் அன்றாட செயற்பாடுகளை எந்தளவு தூரம் பாதித்தது?

பாதிக்கவில்லை ☐
சிறிதளவு ☐
நடுத்தர அளவு ☐
அதிகளவு ☐

6. உங்கள் குழந்தை ரினிடிஸினால் பாதிக்கப்படுவது எப்போதாவது வருடத்தின் சில மாதங்களுக்கு மட்டுப்பபடுத்தப்பட்டதா?

ஆம் ☐
இல்லை ☐

எக்ஸீமா (eczema) தொடர்பான கேள்விகள்

1. உங்கள் குழந்தைக்கு எப்போதாவது, குறைந்தபட்சம் 6 மாத காலங்களுக்கு ஆவர்த்தன முறையில் தொடரும் அரிப்புடன் கூடிய சொறி சிரங்கு(rash) ஏற்பட்டுள்ளதா?

ஆம் ☐
இல்லை ☐

இல்லையாயின் வினா 7 இற்கு செல்க

2. உங்கள் குழந்தைக்கு கடந்த 12 மாதங்களில் அரிப்புடன் கூடிய சொறி சிரங்கு(rash) ஏற்பட்டுள்ளதா?

ஆம் ☐
இல்லை ☐

இல்லையாயின் வினா 7 இற்கு செல்க

3. சொறி சிரங்கானது எப்போதாவது பின்வரும் இடங்களை பாதித்துள்ளதா?
முழங்கை மடிப்பு, முழங்கால் பின்புறம், கனுக்காலின் முன்புறம், இருக்கைபகுதிஇ கழுத்தைச் சுற்றி, கண்கள், காதுகள்?

ஆம் ☐
இல்லை ☐

4. சொறி சிரங்கானது முதன் முதலில் தோன்றிய வயது?

- 2 வயதுக்கு முன் ☐
- 2-4 வயதில் ☐
- 5 அல்லது அதற்கு பின் ☐

5. கடந்த 12 மாதங்களில் எப்போதாவது இந்த சொறி சிரங்கானது முற்றாக நீங்கியதா?

- ஆம் ☐
- இல்லை ☐

6. கடந்த 12 மாதங்களில் எவ்வெப்பொழுது உங்கள் குழந்தை இந்த சொறி சிரங்கு காரணமாக இரவு நேரங்களில் நித்திரையின்றி அவதியுற்றது?

- ஒருபோதுமில்லை ☐
- ஆம் ஆனால் எல்லா கிழமைகளிலும் இல்லை ☐
- ஆம் கிழமைக்கு ஒன்று அல்லது அதற்கு மேற்பட்ட தடவைகள் ☐

7. உங்கள் குழந்தைக்கு எப்போதாவது தோலில் சொறி சிரங்கு ஏற்பட்டதுண்டா?

- ஆம் ☐
- இல்லை ☐
