# Supplementary material for: Prevalence, associated factors, and temporal variation of allergic rhinitis among 13 to 14-year-old adolescents from rural Sri Lanka: An analytical cross-sectional study
Source: Asia Pac Allergy. 2026 Jan 13;16(3):145–51. doi: 10.5415/apallergy.0000000000000252 (PMC13193263; doi:10.5415/apallergy.0000000000000252)
Supplement: Supplementary file 2 [file pa9-16-145-s002.pdf]

Supplementary information 2: Factors associated with allergic rhinitis among 13-14-year-old adolescents without current asthma or eczema in the Anuradhapura municipal council area, Sri Lanka (N=857)

Risk factor	Adolescents with allergic rhinitis		Adolescents without allergic rhinitis		Significance	Unadjusted odds ratio	95% Confidence interval	
	N	%	N	%			Lower	Upper
**Demographic factors**								
Male gender	88	49.44	355	52.28	0.50[b]	1.12	0.81	1.56
Born in Anuradhapura district	162	91.01	616	90.72	0.91[b]	1.04	0.60	1.84
**Housing conditions**								
Having								
cement floor	109	61.24	377	55.52	0.17[b]	1.27	0.90	1.77
marble tile floor	67	37.64	298	43.89	0.13[b]	0.77	0.55	1.08
clay tile roof	30	17.54	150	22.52	0.16[b]	0.73	0.47	1.13
asbestos roof	127	74.27	485	72.82	0.70[b]	1.08	0.73	1.58
concrete roof	09	5.26	22	3.30	0.23[b]	1.63	0.74	3.60
plastered walls	175	98.31	670	98.67	0.72[b]	0.78	0.21	2.93
**Exposure to allergens**								
Having domestic dogs	119	66.85	466	68.63	0.65[b]	0.92	0.65	1.31

Having domestic cats	70	39.33	245	36.08	0.42[b]	1.15	0.82	1.61
Having domestic birds	28	15.73	114	16.79	0.74[b]	0.93	0.59	1.45
Having domestic cows	02	1.12	08	1.18	1.00[c]	0.95	0.20	4.53
Having close contact with animal	93	52.25	362	53.31	0.80[b]	0.96	0.69	1.33
Exposure to smokers at home	16	8.99	58	8.54	0.85[b]	1.06	0.59	1.89
Frequent use of mosquito coils	63	35.39	195	28.72	0.08[b]	1.36	0.96	1.93
Frequent use of kerosene lamps	5	2.81	12	1.77	0.38[b]	1.61	0.56	4.62
Frequent use of incense burners	122	68.54	428	63.03	0.17[b]	1.28	0.90	1.82
Using liquid petroleum gas for cooking	104	58.43	387	57	0.73[b]	1.06	0.76	1.48
Using wood for cooking	57	32.02	214	31.52	0.90[b]	1.02	0.72	1.46
Using electricity for cooking	21	11.8	87	12.81	0.72[b]	0.91	0.55	1.51
Using sawdust for cooking	00	00	02	0.29	1.00[c]	0.79	0.77	0.82
Sleeping on a rubber mattress	160	89.89	634	93.37	0.11[b]	0.63	0.36	1.12
Sleeping on a rubber mixed coir mattress	17	9.55	42	6.19	0.11[b]	1.60	0.88	2.89
Sleeping on a cloth	1	0.56	3	0.44	1.00[c]	1.27	0.13	12.3
**Physical activity**								
Play 4-6 times a week	31	17.42	78	11.49	**0.04**[b]	1.63	1.03	2.56
Play 2-3 times a week	45	25.28	133	19.59	0.65[b]	0.92	0.62	1.34

Play once a week	43	24.16	144	21.21	0.40[b]	1.18	0.80	1.75
Play once a month	12	6.74	56	8.25	0.51[b]	0.80	0.42	1.54
**Dietary habits (Frequent consumption of)**								
pineapple[a]	13	7.3	40	5.89	0.49[b]	1.26	0.66	2.41
tomato[a]	51	28.65	194	28.57	0.98[b]	1.00	0.70	1.45
tuna fish[a]	53	29.78	197	29.01	0.84[b]	1.04	0.72	1.49
king coconut[a]	22	12.36	84	12.37	1.00[b]	1.00	0.61	1.65
sour banana[a]	32	17.98	132	19.44	0.66[b]	0.91	0.60	1.40
ladies' fingers[a]	37	20.79	148	21.8	0.77[b]	0.94	0.63	1.41
curd[a]	18	10.11	60	8.84	0.60[b]	1.16	0.67	2.02
milk powder[a]	62	34.83	207	30.49	0.27[b]	1.22	0.86	1.72
ridge gourd[a]	19	10.67	93	13.7	0.29[b]	0.75	0.45	1.27
Centella[a]	39	21.91	180	26.51	0.21[b]	0.78	0.53	1.15

[a] Frequent consumption is defined as consuming more than twice a week

[b]Chi-square test

[c]Fisher's exact test
