# Supplementary material for: Prevalence, associated factors, and temporal variation of allergic rhinitis among 13 to 14-year-old adolescents from rural Sri Lanka: An analytical cross-sectional study
Source: Asia Pac Allergy. 2026 Jan 13;16(3):145–51. doi: 10.5415/apallergy.0000000000000252 (PMC13193263; doi:10.5415/apallergy.0000000000000252)
Supplement: Supplementary file 3 [file pa9-16-145-s003.pdf]

Supplementary Information 3: All variables included in the multiple regression model for the total number of participants having allergic rhinitis symptoms in each month

Environmental factor (standardized)	Unstandardized coefficient	Standardized coefficient (ß)	T value	Significance	95% CI of unstandardized coefficient	
					**Lower**	**Upper**
Highest monthly temperature	-21.04	-6.42	-2.56	0.034	-40.01	-2.06
Monthly precipitation	30.19	0.92	6.48	0.000	19.45	40.93
Monthly humidity	-20.62	-0.63	-2.60	0.032	-38.93	-2.32
Standardised Mean Monthly Temperature	11.34	0.346	0.87	0.412	-19.38	42.06
Standardised Wind Flow	6.39	0.195	0.77	0.473	-14.04	26.82
Standardised Lowest Monthly Temperature	3.81	0.116	0.39	0.711	-21.16	28.78
Standardised Dew Point	-1.79	-0.055	-0.24	0.821	-22.34	18.77
